# The MAPK Signaling Pathways as a Novel Way in Regulation and Treatment of Parasitic Diseases

**DOI:** 10.3390/diseases7010009

**Published:** 2019-01-17

**Authors:** Yumin Zhao, Weifeng Gui, Fuqiu Niu, Shigui Chong

**Affiliations:** Department of Nursing Teaching and Research, School of Basic Medicine, Guilin Medical University, Guilin 541004, China; zhaoym@lzu.edu.cn (Y.Z.); 17779897914@163.com (W.G.); Niufuqiu@163.com (F.N.)

**Keywords:** MAPK, mitogen-activated protein kinase, transduction pathway, parasite

## Abstract

Few major advances in fighting parasitic diseases have been made in China since the development of new methods for prevention, control, and elimination. However, the proportion of immunocompromised individuals has increased due to the growth of chronic diseases, population aging, and more frequent cases of patients with AIDS and cancer. All these problems can promote development of parasitic infections, which is commonly associated with manipulation of host signaling pathways and the innate immune system. Mitogen-activated protein kinase (MAPK) signaling pathways are evolutionarily conserved in metazoan organisms, which play critical roles in the cell cycle, gene expression, growth, differentiation, apoptosis, and parasite–host interactions. Recent discoveries of the MAPK components involved in activation, regulation, and signal transduction appeared to be promising for the diagnosis, prevention, and treatment of parasitic diseases in the future. This review summarizes the involvement and critical role of the MAPK family in parasitic disease development and maintenance in the host. Moreover, it highlights recent studies concerning the mechanisms and novel drug development for inhibition and regulation of MAPK pathways in order to prevent parasitic disease. In addition, we discuss some antigenic proteins as prospective inhibitory molecules or vaccines for the regulation and control of MAPK signaling involved in parasite physiological activity.

## 1. Introduction

Significant advances have been made in the control and elimination of human parasitic diseases in China since the growth of central and local government attention in developing a range of control programs, as well as improvements in hygienic conditions. Therefore, recent improvements resulted in significant decrease of clinical incidents associated with the hookworms, roundworms, and other soil-transmitted infections. However, the increased prevalence of foodborne parasitic diseases in some provinces of China remains a challenging issue. For instance, the abundance of hydatidosis is still a serious problem in Western China [1]. Furthermore, the growing number of people in the older generation and the increase of chronic diseases resulted in an increase in the number of immunocompromised individuals in the population, which also includes frequent cases of patients with AIDS or cancer [2]. Therefore, the acute problem of parasitic infections is one of the major subjects for health protection and research in China.

Many pathogens target a variety of cell signaling pathways to inhibit or modulate the host’s immune response [3]. Besides strategies to overcome the immune response, pathogens can also utilize cell signaling, including the mitogen-activated protein kinase (MAPK), for the most appropriate cellular response [4].

**Figure 1:**
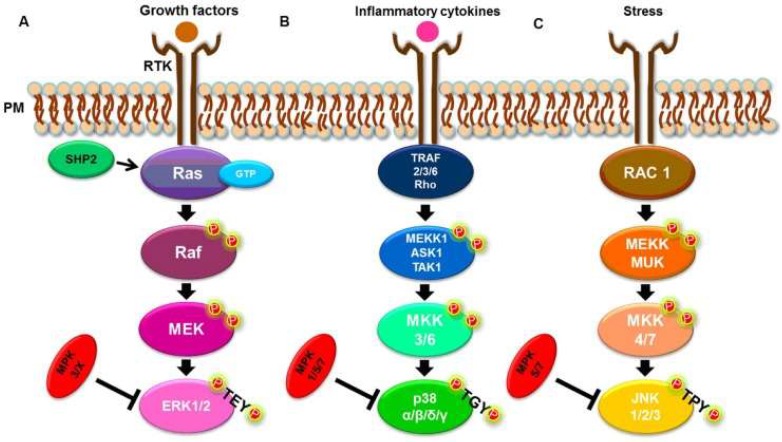
Simplified MAPK signaling pathways. (**A**) ERK1/2 pathway. (**B**) p38 α, β, δ, and γ pathways. (**C**) JNK 1, 2, and 3 pathways [6].

MAPK signaling includes many evolutionary conserved pathways, which are essential in virtually all metazoan organisms [5]. MAPK signal transduction molecules can modulate a variety of crucial functions in both host and parasite cells, such as cell differentiation [6,7], proliferation [8,9], and apoptosis [8,10]. For instance, Erika Pellegrini et al. found that the intracellular parasite *Toxoplasma gondii* has an ability to deliver a protein, GRA24, into cells, which establishes an interaction with a host protein, p38αMAPK(MAPK14). Consequently this interaction results in activation and nuclear translocation of the host kinase, which triggers an inflammatory response [11]. In addition, MAPK is regulated through the activation of three family members: MAPKKK/MEKK/MKKK, MAPKK/MEK/MKK, and MAPK [5]. Therefore, the MAPK pathway is subdivided into three branches: ERK1/2, JNK1/2/3, and p38 MAPK (Figure 1) [6].

Taking in account an important role of MAPK in cell proliferation and survival, many researches are focused on developing novel drugs for eliminating parasites via a specific inhibition of their MAPK pathway. Moreover, a profound study of the mechanism of function and regulation of MAPK signaling could reveal important findings regarding a molecular mechanism of the host–parasite interaction. Therefore, it can be extensively utilized for diagnostic, treatment, and prevention of parasitic diseases.

## 2. Research Directions and MAPK Mechanism of Action in Parasitic Diseases

### 2.1. The MAPK Interplay Between a Parasite and Host

Currently, there are no ideal vaccines or methods for diagnosis or treatment for various parasitic diseases due to the lack of knowledge in molecular levels of manipulated immune response by parasites. Thus, studies are required to increase our understanding of MAPK-dependent mechanisms, which are one of the most important in host–parasite interaction.

Zhang C et al. found that the mechanism of simultaneous activation of cell proliferation and apoptosis in human hepatocytes infected with *Echinococcus multilocularis* [9]. This mechanism is regulated through the activation of ERK1/2 and downstream molecules at the early stage of infection, which results in proliferation of host hepatocytes. Moreover, the damage caused by parasites and related cytokines do not affect hepatocytes during the continuous activation of the pathway. However, consequent activation of JNK and the expression of some specific molecules trigger a growth arrest and induce apoptosis of hepatocytes (Table 1).

Zhou L. et al. found that *Clonorchis sinensis* lysophospholipase A (CsLysoPLA) can activate the PKA-dependent B-Raf/ERK1/2 pathway in mouse macrophages [12]. In their study, it was demonstrated that CsLysoPLA triggered upregulation of IL-25 secretion in macrophages through the MAPK pathway. Therefore, IL-25 can activate hepatic stellate cells (HSCs), resulting in enhanced proliferation of hepatic mesenchymal cells and liver fibrosis (Table 1) [12].

ERK1/2 MAPK activation by *Trypanosoma cruzi* (*T. cruzi*) associated with cellular proliferation in the BeWo trophoblast cell line was shown by Droguett D et al. (Table 1) [7]. Moreover, Hassan et al. have also demonstrated that *T. cruzi* can induce proliferation of vascular smooth muscle cells in the myocardium [13]. In addition, it has also been reported by Bouzahzah et al. that promoted proliferation of hepatic cell via altered activation of ERK1/2 MAPK signaling pathway was induced by *T. cruzi* [14].

Of the most interesting findings about human neutrophils function is a unique form of cell death, netosis, which is associated with granular and cytoplasmic proteins release which entraps and kills microbes. Interestingly, *Leishmania amazonensis* (*L. amazonensis*) can release extracellular traps of neutrophils, which lead to elimination of the parasite. The *L. amazonensis* induced netosis is triggered through the ERK pathway, in which activation takes place downstream of the PI3K cascade independent of AKT and upstream ROS generation (Table 1) [15].

Altogether, *Leishmania* spp. and *T. cruzi* use different strategies for a survival in a host cells after infection. For instance, they evolved to target the MAPK signaling pathways and highjack the immune response of a host cell. Moreover, it can target macrophages, and use host molecules to favor infection process and survival inside host cells (Table 1) [16].

A state-of-the-art research conducted by Quan JH et al. provided a new insight on the intracellular networks of the PI3K/AKT and MAPK signaling cascades for regulating *T. gondii*-induced IL-23 and IL-12 secretion in human monocytic cells (Table 1) [17]. In their work, it was demonstrated that production of IL-23 in *T. gondii*-infected THP-1 cells was regulated mainly by TLR2 and consequently by PI3K and ERK1/2. However, IL-12 production was mainly regulated by TLR4 and then by p38 MAPK and JNK [17].

### 2.2. Detection and Prevention of Parasites at MAPK Level

Similar to an antigen, parasites can trigger the immune response of a host. For instance, the cross-linking reaction protein (CRP) of *Schistosoma mansoni* (*S. mansoni*), especially in female parasites, can specifically react with an antibody against human ERK kinase [18]. Furthermore, Zhao Quan et al. demonstrated that the recombinant MAPK1 protein of *T. gondii* can be potentially utilized as a diagnostic antigen [19].

The recombinant proteins of parasites also have biological functions in stimulating cellular and humoral immunity. Therefore, it can be implemented in developing novel ways for enhancing a host’s immune response without causing tissue damage by utilizing replicated parasite genes responsible for the immunogenic products. As an example, ROP18 and TgMAPK1 gene coding proteins can be utilized as a potential vaccine candidate in *T. gondii*. ROP18 is a newly discovered rhoptry protein with Ser-Thr kinase activity, which is an important regulator of *T. gondii* proliferation in host cells, as well as a key virulence factor. The ability of ROP18 to regulate host immune-related cytokines and inhibit the transcription of host cells by downregulating the MAPK signaling pathway makes him the most appealing vaccine candidate [20]. For instance, cells infected with a type I *T. gondii* display overexpression of ROP38, which consequently inhibits the anti-infection-related genes [21]. Moreover, the expression products of MAPK1 and MAPK2, encoded by genes of *T. gondii*, are closely related to its growth, development, and pathogenicity; they are also considered virulence factors [22]. In addition, bioinformatics analysis of the TgMAPK1 gene structure of *T. gondii* revealed the specific characteristics of its encoded protein. This protein is characterized as immunogenic, soluble, and with multiple epitopes, which can be potentially utilized as vaccine candidate antigens for toxoplasmosis [23].

### 2.3. Treatment of MAPK-Dependent Parasitic Diseases

MAPK signaling plays a crucial role in the process of a parasite and host interaction, alongside the invasion step and proliferation of the parasite. For instance, Song Xinshuai et al. demonstrated the importance of MAPK1 in a parasite and host interaction by constructing MAPK1-deficient *T. gondii*. Therefore, they showed that upon MAPK1 deletion in *T. gondii*, the levels or adhesion, proliferation, and toxicity were significantly reduced (Table 2) [10].

Various studies have indicated the possibility to reduce invasiveness and pathogenicity of parasites through the interference with their normal activity of MAPK signaling pathway. The study of *T. gondii* showed that different components of MAPK pathways display a broad spectrum of effects on the differentiation and proliferation of tachyzoites in host cells. Invalidation of corresponding MAPK molecules in the pathway is one of the important factors in order to reduce the invasion efficiency or proliferation rate of tachyzoites [24]. One of the molecules for controlling invasiveness of parasites is PD98059, which is a specific inhibitor of ERK1/2. The inhibitory effect of PD98059 results in a significant reduction of MEK1 activity, leading to poor levels or even termination of invasiveness [25]. Additionally, a variety of studies have shown that MAPK inhibitor U0126 can also significantly inhibit the invasion of *T. gondii* tachyzoites into host cells by interfering with the sequential release of secretory organelles, such as micronemes, rhoptries, and densegranules [25,26]. Likewise, phospholipaseA2 (PLA2) inhibitors can induce ERK1 or ERK2 to form a dimer, which results in significant reduction of the invasion efficiency of tachyzoites into the host cells. Moreover, a penetration enhancing factor (PEF) also causes obvious changes in the process of tachychite invading host cells. It is noteworthy that U0126 inhibits the cytokines, which is activated by the invasion of *T. gondii*, and causes a destruction of the balance between the host autoimmune response system and *T. gondii*. This molecular mechanism of U0126 action leads to inhibition of the *T. gondii* invasion into the host cells [27].

Unlike *T. gondii*, low concentrations of p38 inhibitors can effectively inhibit the activity of p38 protein kinase in *E. multilocularis* and reduce growth and development of the parasite. However, there is no effect on the activity of p38 protein kinase in human cells. EgRAS, EgRAL, and EgP38 genes of *Echinococcus granulosus* (*E. granulosus*) are potential drug targets, which can be regulated for a treatment of echinococcosis in order to interfere with growth and invasion of *E. granulosu*s [28]. Both SB202190 and SP600125 inhibitors of p38 and JNK, and can trigger apoptosis of *E. granulosus* protoscolices in metacestode through inhibition of MAPK signaling pathway in *E. granulosus* [29].

Chauhan et al. found that DBA (dibenzalacetone) can lead to apoptosis off cells in *Leishmania donovani* (*L. donovani*) via the activation of the MAPK cascades (Table 2) [11].

Zhe Cheng et al. [30] found that EGF-mediated EGFR/ERK signaling pathway promotes germinative cell proliferation in *E. multilocularis* and contributes to larval growth. Targeting the signaling pathways involved in regulating germinative cells may provide a novel therapeutic strategy against echinococcosis and other human cestodiasis (Table 2). In fact, pyridinylimidazoles have already been proved promising compounds with anti-*Echinococcus* activity by specifically targeting the p38 MAPK family [5].

## 3. Conclusions

MAPK signaling pathway is a critical mechanism, which is involved in cell growth, proliferation, and differentiation. Numerous studies focusing on gene cloning, sequence analysis, and functional identification of molecules related to MAPK signaling pathway provided a better understanding about the mechanisms involved in the interaction between parasite and host, which cause various pathological processes. These findings provide novel avenues for development of new methods for prevention, diagnosis, treatment, and clinical application against parasitic diseases. Recently, significant progress has been made in the study of MAPK signaling in parasitic diseases. However, further studies are required for better understanding of the signal molecules and mechanism involved in it. Therefore, it has a great potential in control of parasitic diseases, clinical applications including diagnosis, prevention, and treatment.

## Figures and Tables

**Table 1:** MAPK pathways in host cell infected by parasite.

Host Cell Infected by Parasite	Pathological Importance	The Role of the Pathway	Reference
Mice liver cells infected by *E. multilocularis*	Hepatocyte apoptosis after proliferation as well as damage of liver structure and function.	Early infection: Activation of ERK1/2 contribute to hypatocyte proliferation. Terminal infection: Activation of JNK contribute to hypatocyte apoptosis.	[9]
Mouse macrophage cell line (RAW264.7) cultured and treated with CsLysoPLA	Hepatic fibrosis during *C. sinensis* infection as well as damage of liver structure and function.	PKA-dependent B-Raf/ERK1/2 pathway in mouse macrophages was activated by CsLysoPLA→upregulated production of IL-25→activates HSCs.	[12]
BeWo trophoblastic cell line infected by *T. cruzi*	Cause trophoblast epithelium turnover, which is considered as part of innate immunity.	Participate Bevo trophoblast maintenance and differentiation.	[7]
Neutrophil infected by *L. amazonensis*	Neutrophils netosis can kill parasite.	*L. amazonensis* induce neutrophils netosis through ERK pathway, which acti-vation downstream of PI3Kg and upstream of ROS generation.	[15]
Macrophages infected by *Leishmania* spp. and *T. cruzi*	Highjack the immune response, and, in this manner, promote parasite maintenance in the host.	Parasite target on macrophages MAPK pathways to modulate host immune system and to favor it replication and survival.	[16]
Human THP-1 Cells infected by *T. gondii*	IL-23 and IL-12 regulate both innate and adaptive immunity.	IL-23 production in *T. gondii*-infected THP-1 cells was regulated by ERK1/2. IL-12 production in *T. gondii*-infected THP-1 cells was regulated by p38 MAPK and JNK.	[17]

**Table 2:** MAPK pathways in parasite cell.

Parasite	The Role of the Pathway	Reference
*T. gondii*	Play an important role in tachyzoite invasion and proliferation.	[10]
*E. multilocularis*	Promote growth of metacestodes and germinative cell proliferation.	[30]
*L. donovani*	Leads to apoptotic cell death in parasite.	[11]
